# Unsuspected Cause of Respiratory Distress: Unrecognized Esophageal Foreign Body

**DOI:** 10.1155/2018/6283053

**Published:** 2018-08-19

**Authors:** Naima Baddouh, Lahcen Arjdal, Abdelaziz Raji, Mounir Bourrous

**Affiliations:** ^1^Department of Emergency Unit, Mother and Child Hospital, Mohammed VI University Hospital, Faculty of Medicine, Cadi Ayyad University, Marrakech, Morocco; ^2^Department of Otolaryngology–Head and Neck Surgery, Mohammed VI University Hospital, Faculty of Medicine, Cadi Ayyad University, Marrakech, Morocco

## Abstract

**Summary:**

Foreign bodies in esophagus are avoidable accidents that occur most often in children younger than 3 years. The most common presenting symptoms are dysphagia, drooling, and vomiting. Revelation by respiratory distress is a rare and unusual condition.

**Objective:**

We describe and discuss the case of an esophageal foreign body, in which the patient presented with respiratory distress.

**Case report:**

A two-year-old child was admitted to the emergency department for acute respiratory distress. He had no history of choking episodes or dysphagia. Nevertheless, he was brought by his parents several times for a persistent cough and wheezing that was treated as asthma for a month. Pulmonary examination had revealed polypnea, suprasternal recession, scattered snoring, and diffuse wheeze. As part of his assessment, a chest X-ray was demanded. It had shown, as unexpected, a nonmetallic foreign body in the upper thoracic esophagus. A clothing button was removed by hypopharyngoscopy under sedation without any incident. Subsequent follow-up had not shown any complications related to this episode.

**Conclusion:**

Large esophageal foreign bodies can impinge on the trachea causing upper respiratory tract signs. We alert clinicians on variation in the presentation of foreign body ingestion, and we emphasize the importance of an early diagnosis and management.

## 1. Introduction

The natural inclination of children to explore their environment orally makes the ingestion of foreign bodies (FBs) common, especially in those less than four years [1]. Most foreign bodies ingested get through the digestive tract without clinical manifestations or complications. However, a percentage may have a significant impact on the esophagus and cause typically sialorrhea, odynophagia, and dysphagia. Less frequently, a foreign body (FB) hosted in the esophagus may present with respiratory symptoms, mainly with cough and stridor instead of gastrointestinal symptoms [2]. Revelation by respiratory distress is even rarer [3].

The diagnosis may be delayed leading to several complications especially if the ingestion of the FB is unwitnessed and when the clinician does not consider ingestion of FB as a probable cause of chronic respiratory signs. We report here the case of an esophageal foreign body, in which the patient presented with respiratory distress.

## 2. Case report

A two-year-old boy was brought to the emergency department with respiratory distress without fever. He did not have a pathological perinatal history, nor a personal or family atopy. There was no history of choking episodes or ingestion of a foreign body witnessed by the parents, and he had never experienced dysphagia.

During the last month before admission, he had repeated attacks of cough and wheezing without history of dysphagia or drooling. He was given antibiotics and steroids for a month in response to suspected asthma. However, cough got progressively worse. The appearance of dyspnea had motivated an emergency consultation.

**Figure 1:**
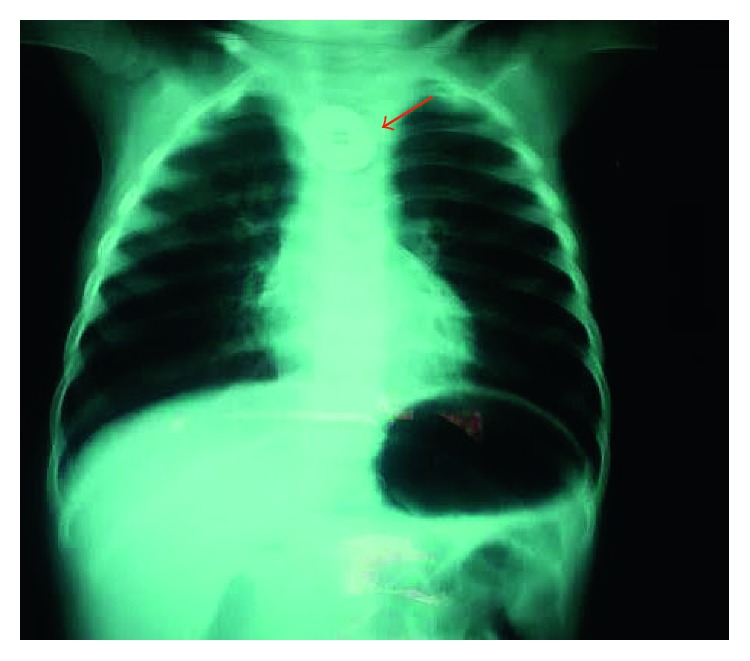
Anteroposterior chest X-ray showing a foreign body in the upper thoracic esophagus.

On examination, the patient was conscious, without cyanosis. He had a temperature of 37.5°C, a heart rate of 130 beats per minute, a respiratory rate of 40 breaths per minute, an oxygen saturation of 93%, and no sign of dehydration or malnutrition. Pulmonary examination revealed suprasternal recession, scattered snoring, and diffuse wheeze. The cardiovascular examination was normal; the rest of the physical examination was unremarkable. Blood tests showed a normal complete blood count and a C-reactive protein of 14 mg/l; nevertheless, an anteroposterior chest X-ray showed a foreign body in the upper region of the mediastinum (Figure 1).

The foreign body was extracted by hypopharyngoscopy under sedation. A clothing button was removed from the upper thoracic esophagus. The mucosa around the button was irregular. There was no obvious stricture or perforation.

Thereafter, the infant was maintained on intravenous fluids, with corticosteroids, to decrease the periesophageal inflammation and was discharged at 24 hours. There were no clinical signs indicative of a perforation, and the following day, he was started on a clear liquid diet. Over the ensuing 1 year, the child has been well and gaining weight satisfactorily. There were no reports of subsequent hospital attendances related to this episode.

## 3. Discussion

Foreign body (FB) ingestion is a frequent and serious problem in children who can be presented with a wide variety of symptoms. It occurs most often in those aged 1 to 3 years because of increasing curiosity and their natural instinct to put everything in the mouth [4].

A foreign body revealed by respiratory signs is not usual; revelation by respiratory distress is even rarer. To our knowledge, very few cases are reported in the literature. Less than 10 cases are published so far [3]. In this case report, the parents did not witness the ingestion of the foreign body, and the boy had shown no obvious drooling.

A chronic FB that is retained in the esophagus more than one week is rare. It presents differently, and the respiratory symptoms are more common than gastrointestinal symptoms [5].

Clinicians should keep in mind that an esophageal FB can lead to atypical symptoms that simulate asthma, croup, bronchitis, or bronchopneumonia. Our patient had a persistent cough that was treated as asthma without improvement.

Most commonly described esophageal FBs are coins, and other ingested objects include toy parts, jewels, batteries, needles, pins, balls, and buttons [1, 6].

Long-standing FBs may have serious consequences such as pneumonia, ulceration, fistula, mediastinitis, and pneumothorax [5]. The most likely mechanism is direct compression of the posterior wall of the trachea by the impacted FB as illustrated by our case. The compressive effect on the trachea is due to the flexible nature and the narrower diameter of the trachea in children [7, 8].

Since, approximately, 63% to 85% of the FBs are radio-opaque, a simple anteroposterior and lateral X-ray of the thorax and neck can confirm the location, the number of FBs ingested, the size, the shape of the object, and the presence of complications. If the FB is radiolucent, the esophagogram can reveal it [5].

Sharp objects have a tendency to get stuck at the level of upper esophagus. In these cases, direct vision or laryngoscopic-aided view could be enough to make the diagnosis and to extract the FB. Esophageal FBs that do not pass to the stomach tend to get impacted at one of the three following levels: the thoracic outlet (70%), mid-esophagus, where the aortic arch and the carina overlap (15%) and at the lower esophageal sphincter (15%) [6].

Rigid endoscopy under general anesthesia is the treatment of choice for esophageal FBs in pediatric. It allows a better view of the FB and uses clamps of different sizes, but in children, it requires a general anesthesia [9]. In view of urgency and the unavailability of rigid digestive endoscopy in our training, an attempt to remove the FB by hypopharyngoscopy under sedation was made in our case. It allowed a successful extraction of the FB without incident despite the localization in the upper thoracic esophagus. We estimate that it can serve as an alternative to save patients in pediatrics in case of emergency and unavailability of rigid endoscopy, especially that it does not require a general anesthesia.

## 4. Conclusion

A chronic esophageal FB can be revealed by nonspecific and unexplained respiratory symptoms, without evidence of dysphagia. The facts that the accident of ingestion was not witnessed, the infant in our case was treated as asthma for one month, until revelation of the FB by a respiratory distress which is a rare complication. Medical staff should be aware of the variation in FB presentation and of the importance of its rapid diagnosis and management. It should be emphasized that protecting children from access to objects that can be swallowed is the best method of preventing FB injestion.
